# EffRes-DrowsyNet: A Novel Hybrid Deep Learning Model Combining EfficientNetB0 and ResNet50 for Driver Drowsiness Detection

**DOI:** 10.3390/s25123711

**Published:** 2025-06-13

**Authors:** Sama Hussein Al-Gburi, Kanar Alaa Al-Sammak, Ion Marghescu, Claudia Cristina Oprea, Ana-Maria Claudia Drăgulinescu, Nayef A. M. Alduais, Khattab M. Ali Alheeti, Nawar Alaa Hussein Al-Sammak

**Affiliations:** 1 Faculty of Electronics, Telecommunications and Information Technology, National University of Science and Technology Politehnica Bucharest, 060042 Bucharest, Romania; ion.marghescu@upb.ro (I.M.); claudia.oprea@upb.ro (C.C.O.); ana.dragulinescu@upb.ro (A.-M.C.D.); 2 Faculty of Computer Science and Information Technology (FSKTM), Universiti Tun Hussein Onn Malaysia (UTHM), Parit Raja 86400, Malaysia; nayef@uthm.edu.my; 3 Department of Computer Networking System, College of Computer Sciences, and Information Technology, University of Anbar, Ramadi 31001, Iraq; co.khattab.alheeti@uoanbar.edu.iq; 4 College of Education for Pure Science, University of Kerbala, Babylon 56001, Iraq; alasadinawar43@gmail.com

**Keywords:** hybrid deep learning, driver drowsiness detection, EfficientNetB0, ResNet50, image classification, real-time video analytics, safety-critical systems, machine learning in transportation, driver monitoring systems, automated fatigue recognition

## Abstract

**Highlights:**

**What are the main findings?**

- EffRes-DrowsyNet, a hybrid deep learning model integrating EfficientNetB0 and ResNet50, achieved high performance across three benchmark datasets: 97.71% accuracy on SUST-DDD, 92.73% on YawDD, and 95.14% on NTHU-DDD.
- The model demonstrated strong generalization and reliability across diverse conditions, maintaining high precision and recall on all datasets.

**What is the implication of the main finding?**

- The hybrid architecture offers an effective balance between computational efficiency and deep visual representation, enabling real-time deployment in driver monitoring systems.
- Its robustness and adaptability make it a promising solution for integration into safety-critical applications, helping to mitigate fatigue-induced accidents in automotive and related domains.

**Abstract:**

Driver drowsiness is a major contributor to road accidents, often resulting from delayed reaction times and impaired cognitive performance. This study introduces EffRes-DrowsyNet, a hybrid deep learning model that integrates the architectural efficiencies of EfficientNetB0 with the deep representational capabilities of ResNet50. The model is designed to detect early signs of driver fatigue through advanced video-based analytics by leveraging both computational scalability and deep feature learning. Extensive experiments were conducted on three benchmark datasets—SUST-DDD, YawDD, and NTHU-DDD—to validate the model’s performance across a range of environmental and demographic variations. EffRes-DrowsyNet achieved 97.71% accuracy, 98.07% precision, and 97.33% recall on the SUST-DDD dataset. On the YawDD dataset, it sustained a high accuracy of 92.73%, while on the NTHU-DDD dataset, it reached 95.14% accuracy, 94.09% precision, and 95.39% recall. These results affirm the model’s superior generalization and classification performance in both controlled and real-world-like settings. The findings underscore the effectiveness of hybrid deep learning models in real-time, safety-critical applications, particularly for automotive driver monitoring systems. Furthermore, EffRes-DrowsyNet’s architecture provides a scalable and adaptable solution that could extend to other attention-critical domains such as industrial machinery operation, aviation, and public safety systems.

## 1. Introduction

Driver drowsiness is a significant risk factor in road traffic accidents, contributing to substantial morbidity and mortality worldwide. The NHTSA (National Highway Traffic Safety Administration) estimates that in 2017, drowsy driving was responsible for 91,000 crashes. Due to the high number of crashes, there were 50,000 injuries and nearly 800 deaths [1]. However, the NHTSA admits that it is hard to determine the precise number of drowsy-driving accidents, injuries, or deaths and that the reported numbers are underestimates [1,2]. According to the EU Mobility and Transport Road Safety Commission, the causes of 10 to 25% of all road accidents in Europe are due to the drowsy state of the driver [3]. Monitoring a driver’s drowsiness is a complex problem involving many behavioral or physiological indicators. Drowsiness is a challenging problem that can lead to road disasters. A sleeping driver is more dangerous on the road than a speeding driver [4]. Drowsiness, even for a short period, can have life-threatening repercussions [5]. The challenge of detecting drowsiness effectively is compounded by the diverse manifestations of fatigue and its fluctuating nature, resulting in vehicular collisions, personal injuries, and even fatalities [6]. Advanced driver monitoring systems (ADMSs) utilizing computer vision have emerged to mitigate road safety issues, including driver drowsiness, by seamlessly integrating with automated control mechanisms to effectively notify drivers of their distracted behaviors [7]. Detecting driver fatigue early in the onset of drowsiness can effectively prevent accidents by providing timely warnings to drivers [8]. Timely interventions that alert drivers can significantly reduce the likelihood of accidents, enhancing road safety for everyone [9].

Recent advancements in video analytics powered by deep learning have opened new avenues for non-intrusive drowsiness detection through continuous visual monitoring of drivers. The use of video datasets for driver drowsiness detection leverages facial recognition technologies and behavioral pattern analysis to identify early signs of fatigue, such as frequent blinking, yawning, and nodding off [10]. The efficacy of these video-based systems in real-world scenarios has been substantiated through extensive research. For instance, a landmark study by Kamble, K. P. [11] demonstrated that deep convolutional neural networks (CNNs) could achieve high accuracy in detecting drowsiness by analyzing sequences of a driver’s facial expressions and head movements, captured through dashboard-mounted cameras. Further research by Yongfeng et al. [12] explored the dynamics of temporal patterns in driver behavior, employing long short-term memory (LSTM) networks to predict drowsiness episodes based on progressive changes observed in video frames. Their findings underscore the potential of integrating temporal data analysis with CNNs to enhance the predictive accuracy of drowsiness detection systems.

The hybrid model proposed in this research aims to harness the strengths of EfficientNetB0 and ResNet50, which are particularly aimed at processing high-dimensional data from video inputs. EfficientNetB0 provides a scalable architecture that adjusts the depth, width, and resolution of the network, making it ideal for handling the varied visual data encountered in different driving environments [13]. Meanwhile, ResNet50’s ability to train very deep networks through its use of residual connections ensures that even subtle features indicative of drowsiness are captured and utilized effectively [14].

This manuscript is structured as follows: Section 2 provides a review of the pertinent literature, delineating the landscape of current research and foundational studies that underpin our focus on hybrid deep learning architectures for driver drowsiness detection. Section 3 details the methodology, describing the datasets utilized, the data preprocessing techniques employed, and the architectural specifics of the EffRes-DrowsyNet model. In Section 4, we discuss the implementation of our proposed model and provide an in-depth analysis of the experimental results. This section also includes a comparative evaluation of the model’s performance against existing benchmarks, highlighting its efficacy and potential for real-world applications. Finally, Section 5 summarizes the conclusions drawn from our study, reflecting on the broader implications of our findings and proposing avenues for future research.

## 2. Related Works

Various studies have proposed different criteria and solutions for detecting driver fatigue and monitoring attention. Over the years, significant research has been conducted to detect drowsiness and alert drivers to reduce accident rates [15,16].

Identifying drowsiness at an early stage is crucial for preventing accidents, and the automation of this process through artificial intelligence enhances the ability to evaluate more cases efficiently and cost-effectively [17]. In their exploration of CNN models for eye state classification, the authors developed a novel CNN model named the 4D model, which demonstrated a high accuracy of 97.53% in detecting drowsiness by analyzing the eye state from the MRL Eye dataset, outperforming other pre-trained models like VGG16 and VGG19.

The authors of [18] highlighted a real-time driver disturbance monitoring method using state-of-the-art CNNs, including InceptionV3, VGG16, and ResNet50, where the ResNet50 model demonstrated the highest accuracy of 93.69%. The study leveraged a unique dataset that includes both side and front views of drivers, which significantly enhanced the performance and real-time efficiency of the system in detecting driver drowsiness.

In [19], efficient methods were proposed to detect driver drowsiness using facial landmarks to monitor blinks and yawns, as well as deep learning techniques employing MobileNet-V2 and ResNet-50V2 to analyze driver activities in real-time. Leveraging transfer learning, the study achieves a notable accuracy of 97%, demonstrating the potential of these methods to enhance doze alert systems and prevent accidents caused by driver fatigue.

The approach in [20] reveals that, in order to combat driver fatigue, researchers have employed artificial intelligence, specifically CNNs, to analyze the state of a driver’s mouth and eyes, calculating features such as the percentage of eye closure (PERCLOS) and yawning frequency (FOM). This method achieved a significant accuracy of 87.5%, underscoring the potential of AI in identifying and mitigating the risks associated with driver fatigue.

The research in [21] leverages a deep learning approach to detect driver drowsiness by analyzing facial landmarks extracted from live camera footage, employing a dataset of 2904 images representing diverse driving conditions. The study achieved a high accuracy rate of 95% by using feature-based cascade classifiers to recognize facial features in real-time scenarios, showcasing an effective continuous monitoring strategy for driver fatigue.

The authors of [22] introduced a method combining a 2D-CNN and an LSTM network to detect driver fatigue, demonstrating a notable accuracy of 95% on the YawDD dataset. The effectiveness of integrating spatial and temporal data through 2D-CNN-LSTM networks highlights its potential for practical applications in automotive safety technologies, significantly outperforming several existing methods.

The research of [23] employed ensemble CNNs and Dlib’s 68 landmark face detectors to analyze early symptoms of drowsy driving by studying facial cues such as eye closure frequency and yawning. The ensemble CNN models showcased remarkable accuracy, achieving 97.4% for eye-related cues and 96.5% for mouth-related cues, demonstrating superior performance over other pre-trained models and offering a promising solution for integrating drowsiness detection systems into vehicles to enhance driver safety.

The study in [24] advances drowsiness detection for long-haul drivers by implementing Dlib-based facial feature detection algorithms, employing both static and adaptive frame threshold methods that utilize the eye closure ratio (ECR) and mouth aperture ratio (MAR) to assess drowsiness levels. The adaptive frame threshold method, in particular, dynamically adjusts the count of consecutive frames that signify drowsiness, achieving a notable accuracy of 98.2%, thus enhancing the precision and reliability of drowsiness detection in real-world conditions.

In response to the increasing need for effective driver drowsiness detection systems to enhance road safety, numerous studies have employed advanced imaging and machine learning techniques. Table 1 summarizes pivotal research from recent years, highlighting the diverse methodologies and outcomes achieved in the detection of driver fatigue using various CNN models.

In contrast to vision-based methods, the study in [25] introduces a hybrid model that leverages electroencephalogram (EEG) signals for drowsiness detection, combining Fast Neighborhood Component Analysis (FNCA) for feature optimization with a Deep Neural Network (DNN) for classification. The proposed approach was evaluated using both the SEED-VIG dataset and a resting-state EEG dataset collected under controlled sleep deprivation, achieving a peak accuracy of 94.29%. By extracting cognitive features from neural activity, the model effectively identifies drowsiness states and demonstrates strong performance in terms of classification accuracy and learning efficiency.

**Table 1:** Recent advances in image and video-based driver drowsiness detection systems.

Reference	Year	Parameters Analyzed	Methodology	Implementation Details	Accuracy	Dataset Used
[26]	2020	Eye and mouth	CNN	GeForce GTX 1080 Ti (NVIDIA, Santa Clara, CA, USA); Python 3.5.2 (Python Software Foundation, Wilmington, DE, USA); Keras 2.2.4 (Chollet F., open source)	93.62%	Driving Image Dataset from Bite Company
[27]	2020	Eye, head, mouth	3D Convolutional Networks	Alienware R17 (Dell, Round Rock, TX, USA); Ubuntu 16.04 LTS (Canonical Ltd., London, UK); 16 GB RAM; 8 GB GPU (Not specified)	97.3%	NTHU-DDD Public
[28]	2020	Eye	FD-NN, TL-VGG16, TL-VGG19	NVIDIA Jetson Nano (NVIDIA, Santa Clara, CA, USA); Near-Infrared camera (Not specified); Custom CNN on Ubuntu (open source)	95–98.15%	Self-prepared ZJU
[29]	2020	Eye and mouth	Mamdani Fuzzy Inference	Not applicable	95.5%	300-W Dataset
[30]	2020	Eye	Multilayer Perceptron, RF, SVM	Not applicable	94.9%	Self-prepared (DROZY Database)
[31]	2020	Respiration (thermal camera)	SVM, KNN	Thermal camera recording at 7.5 FPS	90%, 83%	Self-prepared Thermal Image
[32]	2020	Mouth	Cold and Hot Voxels	Ambient Temp. Control in Lab and Car	71%, 87%	Self-prepared
[33]	2020	Facial features, Head movements	3D CNN	Python 3.6.1, TensorFlow r1.4, reduced video resolution for training	73.9%	NTHU-DDD Public
[34]	2021	Mouth	CNN	Not applicable	99.35%	YawDD, Nthu-DDD, KouBM-DFD
[35]	2021	Eye, head, mouth	SVM	Not applicable	79.84%	NTHU-DDD Public
[36]	2021	Eye and face	Deep-CNN Ensemble	Python 3.6, Jupyter Notebook, Windows 10, Intel Core i5, 8 GB RAM	85%	NTHU-DDD Video
[37]	2022	Eye, head, mouth	CNN and SVM	Not applicable	97.44%	Newly Created: YEC, ABD
[38]	2022	Eye and face	Dual CNN	NVIDIA Xavier, Intel NCS2; 11-62 FPS performance	97.56–98.98%	CEW, ZJU, MRL
[39]	2022	Face	RNN and CNN	Not applicable	60%	UTA-RLDD
[40]	2023	Eye and face	CNN	Not applicable	98.53%	Self-prepared
[41]	2023	Eye and mouth	Dlib’s Haar Cascade	Dlib Toolkit	98%	Self-prepared
[42]	2023	Facial expressions (eyes open, closed, yawning, no yawning)	CNN, VGG16	2900 images including gender, age, head position, illumination	CNN Accuracy 97%; VGG16: 74% Accuracy	Self-prepared dataset
[43]	2024	Face for signs of tiredness	ResNet50	Model trained on Kaggle dataset, test accuracy achieved at epoch 20	95.02%, Loss: 0.1349	Kaggle (Varied Dataset)

## 3. Methodology

**Figure 1:**
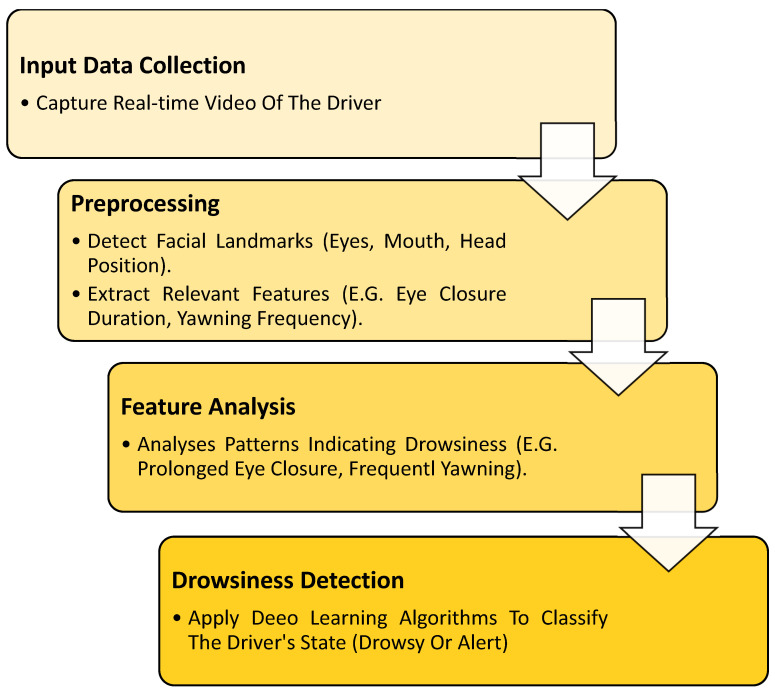
Workflow diagram of the method for driver drowsiness detection.

The method workflow (see Figure 1) follows a structured process to accurately detect the drowsiness of the driver. It first collects data from driver monitoring datasets and then preprocesses it to analyze facial features such as eye and mouth movements. Next, two deep learning models (EfficientNetB0 and ResNet50) extract important details from the image. These features are then combined and optimized to improve accuracy. The system then classifies the driver’s state as drowsy or alert. Finally, if drowsiness is detected, the output triggers a warning, helping to prevent accidents and improve road safety.

### 3.1. Dataset Description

#### 3.1.1. SUST-DDD Dataset

This section discusses the dataset employed, as referenced in [44]. The dataset, known as the SUST-Driver Drowsiness Dataset, is an open-source resource from Sivas University of Science and Technology. It comprises data from 19 participants, with diverse age and gender profiles, recorded predominantly during nighttime and early morning periods to effectively capture their peak drowsiness phases.

Each video is at a resolution of 224 × 224 pixels, segmented into 10 s clips labeled as ‘drowsy’ or ‘alert’.

#### 3.1.2. YawDD Dataset

The YawDD (Yawning Detection Dataset), freely available on IEEE Dataport [45], is an essential resource for research into driver fatigue detection. This dataset comprises over 320 video recordings from 60 participants, showcasing a wide demographic range including gender, age, and ethnicity. Such diversity is crucial to ensure the developed detection models perform effectively across different populations and conditions.

The dataset is thoughtfully segmented into two subsets: the Driver Set, featuring 226 videos in a simulated indoor environment, and the InCar Set, with 94 videos captured in real driving conditions. This setup enables researchers to refine detection algorithms in a controlled environment before assessing their efficacy in more variable, real-world scenarios. Each video in the dataset is meticulously annotated for instances of yawning and other facial expressions related to drowsiness, providing detailed data for training machine learning models with high precision. The variation in video lengths further facilitates the study of both short-term and long-term indicators of fatigue, supporting the development of comprehensive predictive models.

The choice to use the YawDD was driven by its accessibility and the depth of its data, which not only aids in developing sophisticated algorithms but also allows for these models to be benchmarked under simulated and actual conditions. The integration of these models into advanced driver-assistance systems (ADASs) could significantly enhance road safety by mitigating fatigue-related accidents. Therefore, the YawDD dataset is an invaluable asset for advancing research in driver fatigue detection and improving safety measures within the automotive industry.

#### 3.1.3. NTHU-DDD Dataset

The NTHU Driver Drowsiness Detection (NTHU-DDD) dataset [46], developed by National Tsing Hua University, is a publicly available video dataset designed for the evaluation of vision-based driver drowsiness detection systems. It comprises recordings of 36 subjects under various real-world driving conditions, including different lighting environments (daytime, nighttime with and without cabin lighting) and a range of appearances, such as subjects wearing glasses and sunglasses. This diversity enhances the dataset’s applicability to practical scenarios.

Each video sequence is annotated with one of six predefined states relevant to drowsiness detection: Normal, Yawning, Talking, Slow Blink, Closed Eyes, and Sleeping. These annotations enable both binary classification (alert vs. drowsy) and multi-class detection frameworks. The videos are recorded at a resolution of 640 × 480 pixels and a frame rate of 30 frames per second, with both RGB and grayscale versions available.

The dataset is widely used in the academic community due to its comprehensive labeling, subject variability, and simulation of realistic driver behavior. It serves as a robust benchmark for developing and validating machine learning and deep learning models in the domain of driver state monitoring.

### 3.2. Data Preprocessing and Augmentation

#### 3.2.1. Facial Landmark Detection for Drowsiness and Non-Drowsiness

In our study, we employed Dlib’s Histogram of Oriented Gradients (HOG)-based face detection and the 68-point facial landmark prediction model to analyze video frames from the SUST-DDD and YawDD datasets. The choice of Dlib’s HOG-based detector was based on its robust performance across diverse lighting conditions and facial orientations, crucial for the varied environmental settings in these datasets. This detector excels in both controlled and dynamic scenarios, making it highly suitable for our analysis.

**Figure 2:**
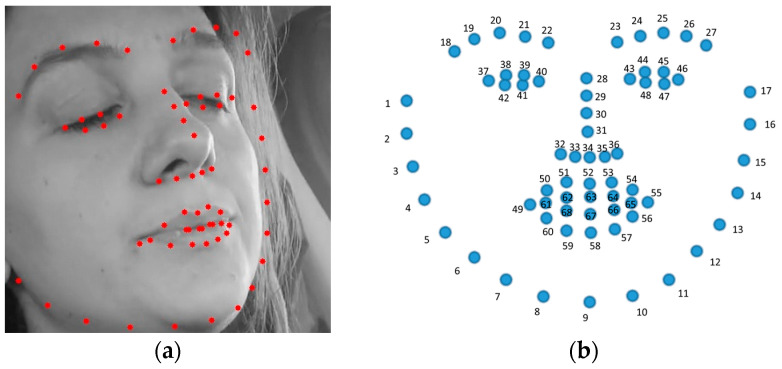
Identification of facial landmarks using Dlib. (**a**) Facial landmarks. (**b**) The position and order of 68 points on the face [47].

Additionally, we utilized the 68-point facial landmark predictor for its precise detection of critical facial points necessary for calculating the eye aspect ratio (EAR) and mouth aspect ratio (MAR). These metrics are vital for quantifying drowsiness indicators such as eye closure and mouth movement. The precision of this landmark detection is fundamental to the accuracy and reliability of our fatigue assessment, ensuring that our methodology adheres to rigorous academic standards and is applicable for enhancing real-world drowsiness detection systems, as shown in Figure 2.

#### 3.2.2. Aspect Ratio Calculation

We computed specific metrics that help detect key fatigue-related behaviors:

- **Eye Aspect Ratio (EAR)**

EAR is calculated to determine whether eyes are open or closed. This is crucial for detecting drowsiness. The eye aspect ratio (EAR) is computed to quantify the vertical eye closure, which is a significant indicator of drowsiness in drivers. The EAR is calculated using Equation (1) as follows [48]:

(1) $${EAR}_{left}=\frac{\left\|P_{38}\right.-\left.P_{42}\right\|+\left\|P_{39}\right.-\left.P_{41}\right\|}{2\left\|P_{37}\right.-\left.P_{40}\right\|}$$

Similarly, the right eye EAR is given by Equation (2) as follows:

(2) $${EAR}_{right}=\frac{\left\|P_{44}\right.-\left.P_{48}\right\|+\left\|P_{45}\right.-\left.P_{47}\right\|}{2\left\|P_{43}\right.-\left.P_{46}\right\|}$$

where

$P_{37}$, $P_{38}$, …, $P_{42\;}$ (for the left eye) and $P_{43}$, $P_{44}$, …, $P_{48\;}$ (for the right eye) are the 2D facial landmark positions that correspond to the key points of a human eye, typically obtained from a facial landmark detection model. $P_{1}$ and $P_{4}$ represent the horizontal corner points of the eye.

$P_{37}$, $P_{40}$ (left eye), as well as $P_{43}$, $P_{46}$ (right eye), represent the horizontal corner points of the eye.

$P_{38}$, $P_{39}$, $P_{41}$ (left eye) and $P_{44}$, $P_{45}$, $P_{47}$ (right eye) represent the vertical boundary points of the eye.

||$P_{i}{-P}_{j}$|| denotes the Euclidean distance between the points $P_{i}{-P}_{j}$.

In this approach, the eye aspect ratio (EAR) threshold is dynamically calculated for each video based on its unique data, enhancing the precision of drowsiness detection. If the EAR falls below this adaptive threshold, it indicates closed eyes, suggesting drowsiness; values above the threshold indicate open eyes. This method ensures that the detection adapts to variations in individual videos, improving the reliability of the analysis.

- **Mouth Aspect Ratio (MAR)**

The mouth aspect ratio (MAR) is used to detect whether a person is yawning or not [49]. The EAR is calculated using Equation (3) as follows:

(3) $$MAR=\frac{\left\|P_{62}\right.-\left.P_{66}\right\|+\left\|P_{63}\right.-\left.P_{65}\right\|}{2\left\|P_{61}\right.-\left.P_{64}\right\|}$$

where

$P_{61}{,\;P}_{62}$, …, $P_{66}$ are the key points on the mouth from a facial landmark detection system.

$P_{61}$ and $P_{64}$ represent the mouth corners (left and right, respectively).

$P_{62},\;P_{63}$ and $P_{65},\;P_{66}$ represent points along the upper and lower lip, respectively, providing vertical measurements for the mouth opening.

$\left\|P_{i}\right.-\left.P_{j}\right\|$ denotes the Euclidean distance between the points $P_{i}$ and $P_{j}$.

Similarly to EAR, the mouth aspect ratio (MAR) is calculated using vertical and horizontal distances between specific mouth landmarks. This ratio is dynamically determined for each video, enhancing detection accuracy. If MAR exceeds the adaptive threshold, yawning is detected, suggesting potential drowsiness. Values below the threshold indicate no yawning. This method adapts to variations in individual videos, ensuring reliable and precise yawning detection.

#### 3.2.3. Feature Fusion and Landmark Accuracy in Driver Drowsiness Detection

Since EffRes-DrowsyNet adopts a hybrid approach that combines handcrafted visual features, specifically the EAR and MAR, with deep features extracted by EfficientNetB0 and ResNet50, the model benefits from both low-level geometric cues and high-level semantic representations, improving its reliability in detecting drowsiness under diverse lighting and facial conditions.

The effectiveness of EAR and MAR depends on the accurate localization of key facial landmarks. In this work, the Dlib 68-point model was adopted due to its reliability in frontal face analysis. However, landmark accuracy can degrade under real-world conditions such as head pose variation, occlusions (e.g., eyeglasses, hand gestures), and variable lighting. These factors may distort EAR and MAR calculations, potentially leading to false classifications.

To mitigate such risks, the following strategies were incorporated:

- Facial alignment was applied to normalize orientation and scale.
- Region of interest (ROI) extraction focused attention on the eye and mouth regions.
- Temporal smoothing of averaged EAR and MAR values across sequential frames to reduce the effect of transient noise and natural blinks.

Importantly, the model does not classify drowsiness based on isolated frame-level changes. Instead, it learns behavioral patterns over time, such as sustained eye closure or repeated yawning. These temporal descriptors are fused with deep visual features, forming a unified representation for final classification. This approach allows the model to distinguish between typical blinking and fatigue-induced states.

While the current method improves robustness, persistent landmark inaccuracies may still affect performance. As part of future work, we plan to explore confidence-based keypoint filtering and fully end-to-end architectures that reduce reliance on explicit landmark measurements while maintaining behavioral interpretability.

#### 3.2.4. Frame Preprocessing

**Figure 3:**
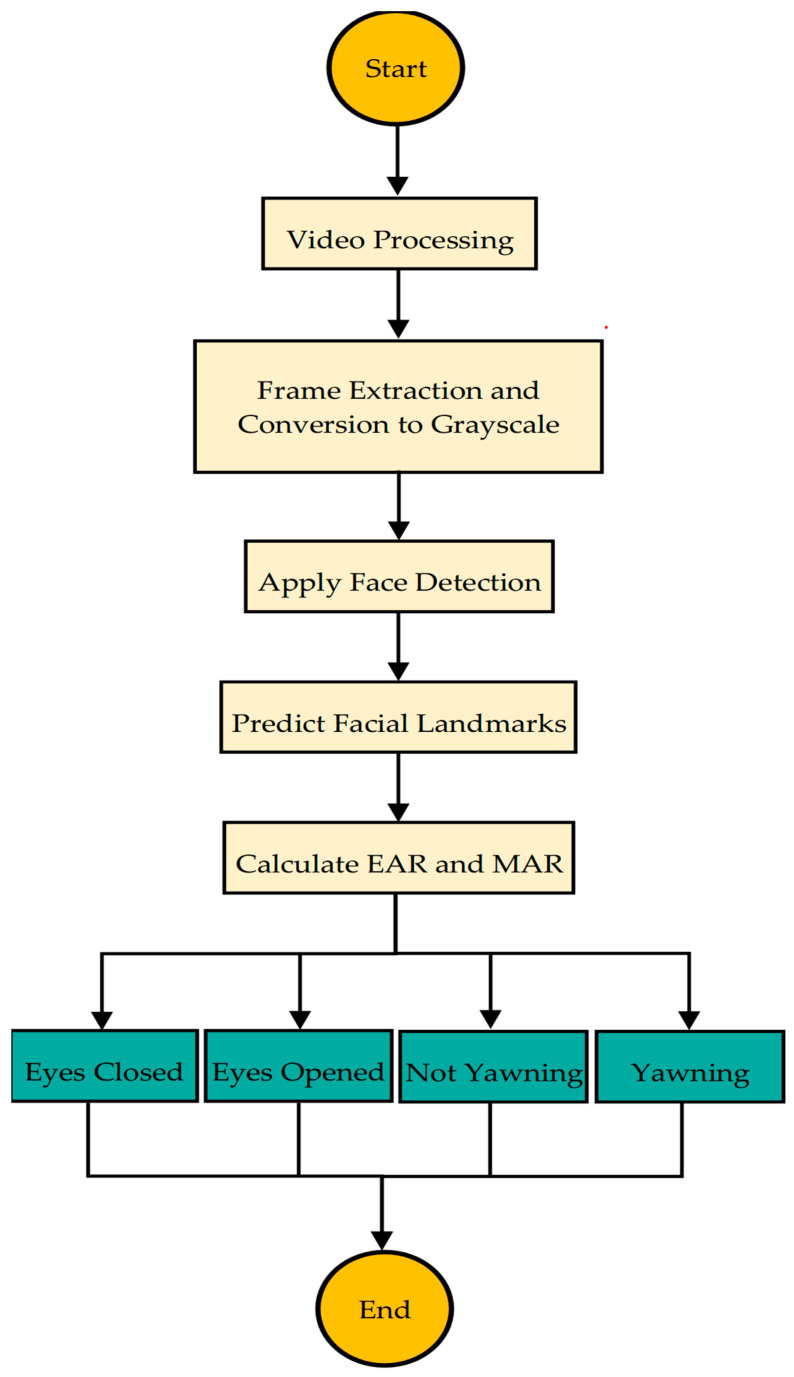
Workflow diagram illustrating the automated video processing pipeline for drowsiness detection.

To prepare the face area for analysis, the video frame undergoes several preprocessing steps, as shown in Figure 3.

First, the bounding box is used to detect and crop faces. Then, the extracted face is resized to 224 × 224 pixels and converted to grayscale to ensure consistent lighting conditions. To further improve visibility, CLAHE (Contrast Limited Adaptive Histogram Equalization) was applied [50], which helped to standardize lighting variations. Finally, pixel values are scaled between 0 and 1, ensuring standardized inputs for the next stage of processing.

#### 3.2.5. Video Processing and Event Detection

Videos have been analyzed frame by frame, and EAR and MAR were used to detect specific events.

**Figure 4:**
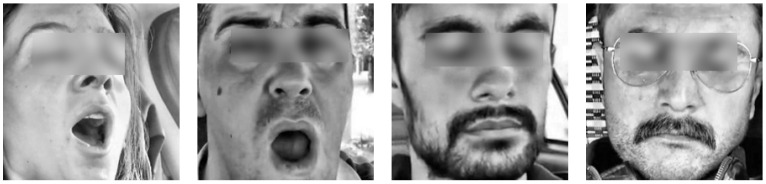
Samples of drowsiness output frames extracted from video dataset.

- Drowsiness Video Processing:

Eyes are considered closed if the EAR falls below the defined threshold. At this stage, all video files in the drowsiness dataset were processed, detecting events of eye closure and yawning. The target frames for each video were saved. Figure 4 illustrates the samples of the detected drowsiness frames.

**Figure 5:**
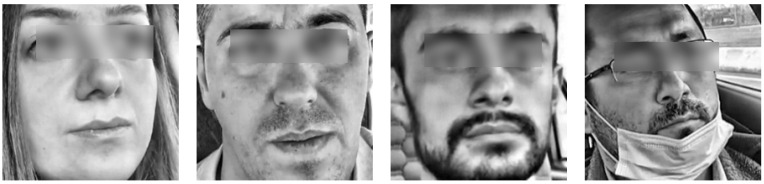
Samples of non-drowsiness output frames extracted from the video dataset.

- Non-Drowsiness Video Processing:

Eyes are considered open if the EAR exceeds the threshold, indicating the subject is not drowsy. The non-drowsiness dataset was processed to detect open eyes and non-yawning states and to save the respective frames. Figure 5 illustrates the samples of the detected non-drowsiness frames.

These two complementary algorithms represent the initial step in the preprocessing pipeline for detecting states of drowsiness and alertness based on facial cues. The algorithms efficiently analyze video frames to detect signs of fatigue, such as eye closure and yawning, as well as indicators of alertness like open eyes and the absence of yawning. By processing both datasets of drowsy and non-drowsy videos, the algorithms extract relevant frames that highlight these behaviors. This preprocessing stage is crucial for further analysis in applications such as driver drowsiness detection and real-time safety monitoring systems. This foundational work sets the stage for deeper analysis and integration into broader systems intended to enhance safety and performance monitoring, as shown in Figure 3.

The automated detection of drowsiness and alertness in video streams algorithm employs a sequence of computational steps to evaluate states of alertness and drowsiness from video inputs. Figure 4 illustrates the comprehensive workflow of this process, utilizing advanced facial detection and image processing techniques to analyze real-time video streams. Following this visual overview, Table 2 succinctly summarizes the essential steps of the algorithm, detailing the initialization of detection systems, configuration of detection metrics, and the processing routines involved. This structured approach ensures that the algorithm systematically identifies and classifies visual cues related to the user’s state, facilitating robust and reliable assessments across diverse settings.

#### 3.2.6. Standardization of Frame Count Across Video Datasets

In video processing and machine learning, it is imperative to maintain a consistent number of frames per video sequence to ensure uniformity in data input, which is crucial for the effective training of neural networks. Algorithm 1 is meticulously designed to normalize the frame count across video sequences in a dataset. This normalization is essential for maintaining consistency in training sessions, particularly in computer vision applications where the input dimensionality must be uniform across all data samples.
**Algorithm 1: *Frame Count Normalization For Video Datasets***
**Input:**

Input directory containing video frame folders, output directory, and desired number of frames per video.

**Initialize Categories:** Check and create the output directory if it does not exist.
**Process:**

**For Each Video Directory in the Input Directory:**Retrieve and Sort Frames:○Collect all .jpg frames from the current video directory and sort them to maintain temporal order.Check Frame Availability:○If no frames are present, log the issue and proceed to the next video directory.Adjust Frame Count:○Excess Frames:▪If the number of frames exceeds the target, select frames evenly using linear interpolation.○Insufficient Frames:▪If the number of frames is less than the target, replicate existing frames systematically until the target count is reached.○Save Processed Frames:▪Store the selected or replicated frames in a new subdirectory within the output directory.
**Output:**

Indicate successful normalization of frame counts for all processed directories.
**End Algorithm**

**Figure 6:**
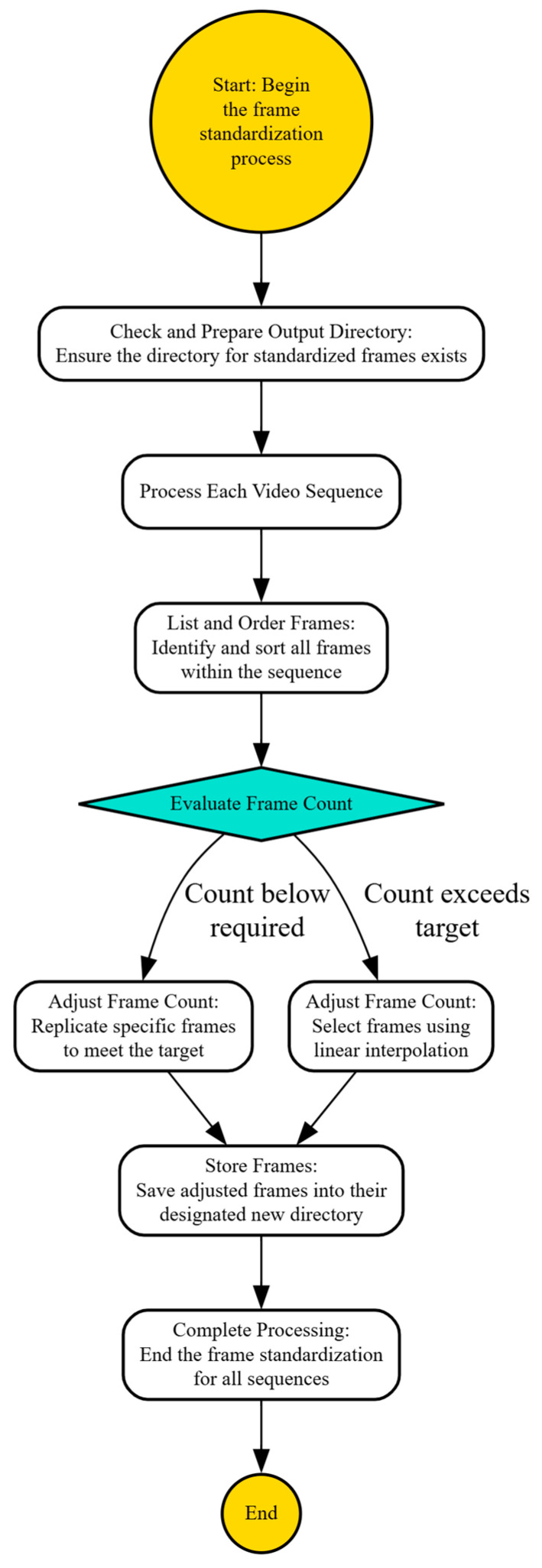
Flowchart depicting the process of standardizing frame counts in video sequences.

Figure 6 represents the systematic approach to ensure each video sequence contains a uniform number of frames for consistent input to machine learning models, involving frame assessment, selection or replication, and systematic storage.

**Figure 7:**
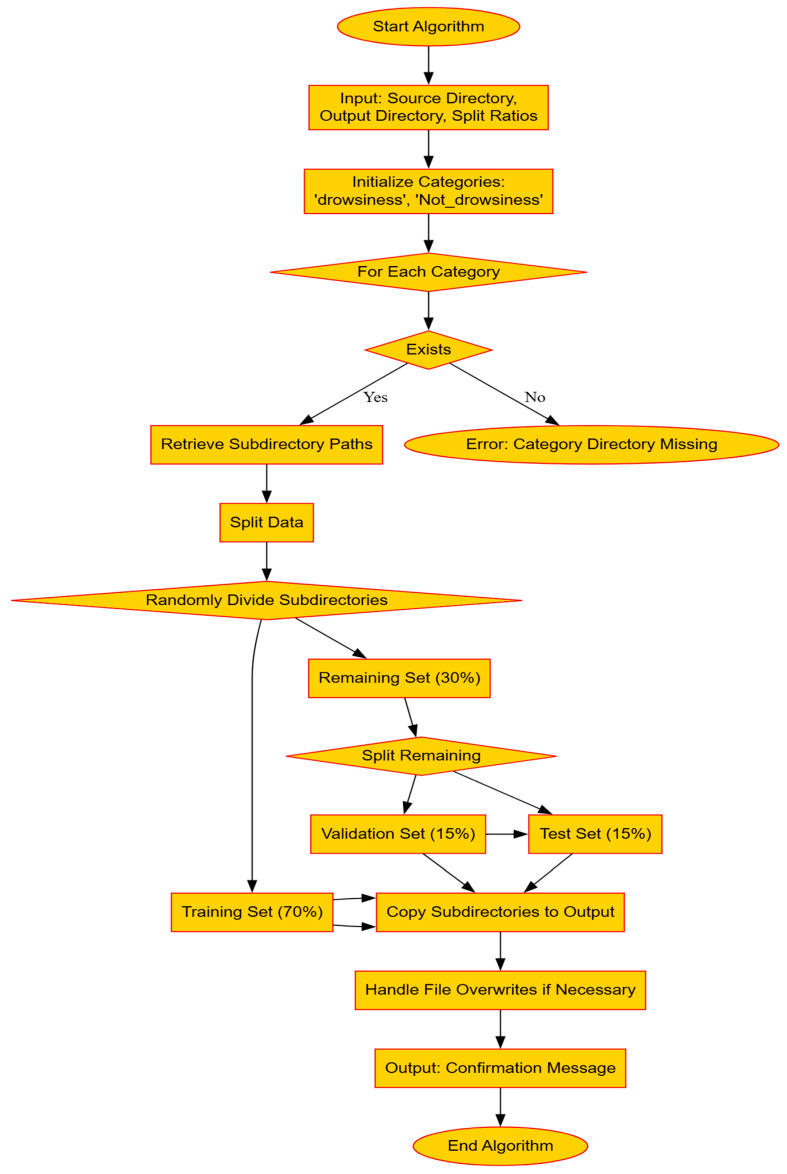
Flowchart of the data splitting process.

The automated organization of video data into distinct training, validation, and testing sets is a critical initial step in the development and evaluation of machine learning models. This process begins by verifying the existence of categorized directories, such as ‘Drowsiness’ and ‘Not Drowsiness’, within a specified source directory. Each category is systematically processed to enumerate all contained directories, representing individual data points. These directories are then randomly allocated to ensure a balanced distribution—70% are assigned to the training set, and the remaining 30% are equally split between validation and testing sets [51]. Such meticulous preparation is essential for unbiased model training and is visually depicted in Figure 7. This structured approach not only streamlines data setup but also enhances the efficiency of subsequent model training and validation tasks.

### 3.3. Model Architecture and Configuration

In this section, we will discuss the architecture of the hybrid model, which leverages two state-of-the-art pre-trained models, EfficientNetB0 [52] and ResNet50 [53], to enhance performance. The architecture focuses on extracting features from these powerful models, concatenating them, and applying regularization to reduce overfitting.

#### 3.3.1. Individual Models

The organized framework consists of two CNNs that include EfficientNetB0 and ResNet50. Both of these networks have been pretrained on the ImageNet dataset. These networks were selected based on the previous image classification documents.

EfficientNet is the name of different models which are designed to have high accuracy with minimum computation. It is an optimized, lightweight model with excellent performance as a feature extractor. The depth, thickness, and resolution scales of the model are all evenly scaled, making it suitable for picture classification tasks falling within an acceptable computation range.

ResNet, also known as Residual Networks, is characterized by a deep architecture containing residual connections, which has been professionalized to eliminate the vanishing gradient problem, thus assisting very deep networks. ResNet50 is a 50-layer architecture model and is specifically very efficient while extracting deep hierarchical features from images.

The hybrid model leverages the convolutional base of two pre-trained architectures (EfficientNetB0 and ResNet50), where the fully connected top layer is excluded (include top = False). These foundations are pre-trained on the ImageNet dataset and essentially capture general visual features such as edges, textures, and patterns. The features extracted from the two models are concatenated, enabling the fusion of complementary representations to improve downstream task performance.

#### 3.3.2. Feature Concatenation and Regularization

After extracting features from both models, the outputs of EfficientNetB0 and ResNet50 are passed through Global Average Pooling layers to reduce their spatial dimensions. This process condenses the high-dimensional feature maps into lower-dimensional feature vectors that retain key information.

The resulting vectors are then concatenated, creating a unified representation that leverages EfficientNetB0’s computational efficiency and ResNet50’s deep feature extraction capacity. To enhance training stability and mitigate internal covariate shift, batch normalization is applied. A dropout layer (rate = 0.3) follows to reduce overfitting and improve generalization.

**Figure 8:**
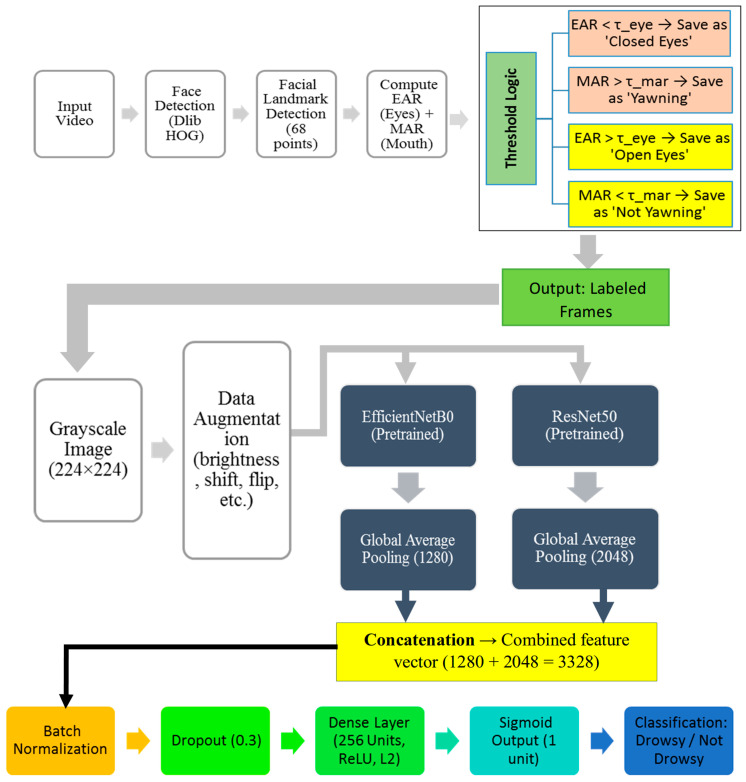
Unified reference model showing the integration of handcrafted EAR/MAR-based behavioral cues with the dual-stream CNN architecture (EfficientNetB0 + ResNet50) in the proposed EffRes-DrowsyNet framework.

Next, a dense layer with 256 units and L2 regularization is used to refine the combined features while penalizing overly large weights. Finally, a sigmoid-activated output layer performs binary classification, predicting whether the driver is drowsy or alert. The overall hybrid architecture is illustrated in Figure 8.

To improve the interpretability and effectiveness of the model, we developed a hybrid channel that combines traditional behavioral cues with deep learning-based visual analytics. As shown in Figure 8, our method first extracts the EAR and MAR from the facial landmarks detected using Dlib. These features are used as key indicators of the driver’s drowsiness, such as closing eyes and yawning, and are used to selectively mark frames based on predefined thresholds. The labeled images are then passed to the dual-stream convolutional models EfficientNetB0 and ResNet50, each of which is pre-trained on ImageNet to extract robust spatial features. Global average pooling is applied to two feature maps, and the resulting vectors are connected to form a unified representation. This hybrid architecture enables the model to learn from domain-specific manual features and depth vision patterns, ultimately improving the accuracy and reliability of drowsiness detection in a variety of real-world scenarios.

#### 3.3.3. Model Architecture

The proposed model architecture uses EfficientNetB0 and ResNet50 as feature generators by removing their classification layers and retaining only the feature extraction components. These networks are pre-trained and effective for image classification, and their extracted features are utilized in a broader system for further processing. Extracted features are subsequently integrated and undergo additional layers for further concentration optimization towards the target task:

**Feature processing.** During the concatenation of the features, the pooling of the global average was performed to minimize spatial dimensionality, and batch normalization and dropout layers were added to prevent overfitting and increase generalization capabilities.

**Dense Layer and Regularization.** Several features are concentrated and compressed by L2 dense layer regularization to the required size, thus enabling binary classification.

**Output Layer.** The final layer, which is the one preceding the output, is a dense layer that has the sigmoid activation function, providing a probability score for the target class.

This architecture is designed to efficiently leverage the general image representations captured in the pre-trained models, while the added layers enable the model to learn task-specific details for binary classification.

#### 3.3.4. Compilation and Training

The model is compiled using the Adam optimizer, chosen for its adaptive learning rate, which accelerates convergence by adjusting the learning rate dynamically for each parameter. Binary cross-entropy is used as the loss function, appropriate for binary classification tasks, as it measures the discrepancy between predicted probabilities and true class labels. Model performance is evaluated throughout training using accuracy, precision, and recall metrics, providing a comprehensive view of its predictive quality.

To enhance the training process, four callback functions are implemented. Thus, EarlyStopping monitors validation loss, halting training when improvement ceases and restoring the best model state to avoid overfitting. Further, ReduceLROnPlateau decreases the learning rate when the validation loss plateaus, facilitating continued model refinement. ModelCheckpoin saves the model with the highest validation accuracy, ensuring the best version is preserved for deployment. TensorBoard visualizes training progress, offering insights into the evolution of loss and accuracy metrics, aiding in identifying overfitting or stagnation.

#### 3.3.5. Fine-Tuning Strategy

An important stage of the training is fine-tuning, as it enables the model to use the pre-trained weights properly. Initially, the mode was to freeze the first hundred layers of EfficientNetB0 and ResNet50, multiclass classifiers built on ImageNet features, that were determined to be helpful. With this approach, lower layers drop essential task-agnostic features while higher layers specialize in the features of the target dataset. After the training on the new top layers, their own layers are unfrozen step by step, and the entire model is again trained with a lower learning rate to ensure only small changes are made to the task while retaining useful features from the pre-trained model.

#### 3.3.6. Data Preparation and Augmentation

Data preprocessing and augmentation are integral to model enhancement. To achieve this objective, the ImageDataGenerator class was applied to conduct on-the-fly data enhancements by performing several transformations, such as rotations, shears, shifts, zooms, horizontal flips, and rescaling pixel values, which provided variation. These augmentations help improve the training data and allow the model to perform well on new and unexposed data.

#### 3.3.7. Training Execution and Evaluation

For a certain number of epochs, the model is systematically trained using the specified batch sizes for the training and validation sets. Data generators streamline the data feeding process, allowing efficient utilization of memory and computational resources. After training, the model undergoes rigorous evaluation on a separate test dataset to assess its real-world applicability. This post-training evaluation provides critical insights into the model’s effectiveness and potential limitations, ensuring it meets the performance standards required for the intended applications.

#### 3.3.8. Implementation Overview

TensorFlow and Keras are utilized to implement the proposed hybrid model, with Keras serving as the high-level API to support modular and flexible model construction. This comprehensive training, architectural, and implementation strategy integrates efficient feature extraction, targeted regularization, adaptive fine-tuning, and detailed evaluation to develop a high-performing model capable of addressing complex image classification challenges in computer vision. The overall process is outlined systematically in the following pseudocode (Algorithm 2), which details each stage from data preparation to final model assessment.
**Algorithm 2: *EffRes-DrowsyNet—Hybrid Drowsiness Detection Model*** 
**Input:**

Video stream ***V***EAR threshold $T_{eye}$MAR threshold $T_{mouth}$Pretrained models: EfficientNetB0, ResNet50Max_Epochs = ***N***, Batch_Size = ***B***
**Output:**

Predicted label: *Drowsy/Not Drowsy*
**Process:**

(1)***Preprocessing and Labeling:***○For each frame ***F*** in video ***V***:Detect face using Dlib HOG detectorPredict 68 facial landmarksCompute EAR and MARLabel−If EAR $<\;T_{eye}$: Eyes = *Closed*−Else: Eyes = *Open*−If MAR $>\;T_{mouth}$: Mouth = *Yawning*−Else: Mouth = *Not Yawning*Assign final frame ***F*** label:−If Eyes = *Closed* or Mouth = *Yawning*: Label = *Drowsy*−Else: Label = *Not Drowsy*(2)***Data Preparation***○Convert all labeled frames to grayscale (224 × 224)○Apply data augmentation: brightness shift, rotation, flipping, etc.○Split dataset into training, validation, and test sets(3)***Hybrid CNN Model Construction (EffRes-DrowsyNet)*** ○Input: Grayscale image $I\;\in \;R^{224\times 224\times 1}$○EfficientPath ← EfficientNetB0 (Replicated as 3 channels)○ResNetPath ← ResNet50 (Replicated as 3 channels)○FeatureEffNet ← GlobalAveragePooling(EfficientPath)   // Output: 1280-D○FeatureResNet ← GlobalAveragePooling(ResNetPath)    // Output: 2048-D○FeatureVector ← Concatenate(FeatureEffNet, FeatureResNet) // 3328-D○***x*** ← BatchNormalization(FeatureVector)○***x*** ← Dropout (***x***, rate = 0.3)○***x*** ← Dense (***x***, units = 256, activation = ‘ReLU’, regularizer = L2)○Output ← Dense (x, units = 1, activation = ‘Sigmoid’)(4)***Training Phase*** ○Compile model with Binary Cross-Entropy loss and Adam optimizer○Train model for max ***N*** epochs or until early stopping triggers○Monitor validation loss for learning rate reduction and overfitting(5)***Inference*** ○For each test video:Preprocess as abovePass through the trained modelPredict label = Drowsy or Not Drowsy based on sigmoid threshold
**End Algorithm**

## 4. Results and Discussion

In this section, we will evaluate the performance of the novel hybrid deep learning model developed by integrating two well-known CNN architectures, EfficientNetB0 and ResNet50. The primary motivation behind this approach was to harness the distinct features and strengths of both architectures to improve the model’s ability to generalize and accurately classify images in a binary classification setting. The combination of these models aimed to capture a broad spectrum of features, ranging from basic to complex patterns, thereby enhancing the model’s predictive performance on complex datasets.

### 4.1. Performance Evaluation of EFFRES-DrowsyNet Across Various Training Epochs on the SUST-DDD Dataset

This section presents a detailed analysis of the experimental results obtained from a model configuration across different training epochs using the SUST-DDD dataset [44]. The objective of these experiments was to investigate the model’s performance over varying numbers of epochs while incorporating early stopping in some cases. Performance was evaluated using four primary metrics: test accuracy, test precision, test recall, and test loss. The goal of this analysis is to compare the effects of different training durations on model effectiveness and identify the most robust experimental setup for optimal performance.

Table 3 illustrates the model’s performance under different experimental setups, varying the number of training epochs. The results presented correspond to distinct executions of the model, each configured with a unique stopping criterion or total number of epochs. The table provides insights into how training duration influences key performance metrics: test accuracy, test precision, test recall, and test loss.

**Figure 9:**
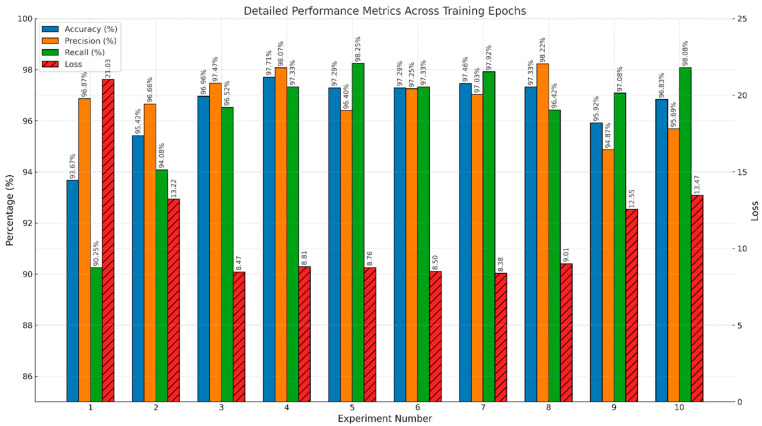
Evaluation of a novel hybrid model performance across training epochs.

Figure 9 delineates the impact of varying training epochs on key performance metrics—accuracy, precision, recall, and loss—across ten distinct experimental setups.

This bar chart precisely quantifies the impact of different training periods on the model’s performance, represented by the metrics of test accuracy, precision, recall, and loss. The dataset encompasses results from ten experiments, each employing a specific number of training epochs, ranging from 10 to 100. A variety of these experiments incorporate early stopping criteria to optimize the training process and mitigate the risk of overfitting. Metrics such as accuracy, precision, and recall are plotted against the primary *y*-axis in percentage terms, while loss is measured on a secondary *y*-axis to maintain scale integrity and improve interpretability. Each metric is marked with vertical labels directly above the bars, ensuring that precise values are readily accessible for detailed analysis. This graphical representation is important in highlighting the relationship between training duration and model efficacy, providing insights into optimal training strategies that balance model accuracy and computational efficiency. The chart supports discussions on effective training methodologies for enhancing model performance in practical deployment scenarios.

#### 4.1.1. Optimal Epochs and Generalization

While deep learning models typically improve in accuracy with increased training epochs, our focus was on identifying the optimal stopping point to balance performance with computational efficiency. As shown in Table 3, Experiment 4, which applied early stopping at 25 epochs, achieved the highest metrics—97.71% accuracy, 98.07% precision, and 97.33% recall—with minimal test loss (0.0881). This indicates that the model had effectively learned the relevant features for drowsiness detection within a relatively short training window, avoiding unnecessary computational costs.

This result is particularly significant for real-time applications like in-vehicle driver monitoring systems, where fast convergence and minimal overfitting are essential. Experiments beyond 30 epochs showed only marginal improvements or even slight degradation in generalization (as reflected by increased loss), reinforcing the effectiveness of early stopping as a practical training strategy.

#### 4.1.2. Impact of Early Stopping

The use of early stopping proved critical in identifying the point at which the model generalizes best without overfitting. Among all experiments, Experiment 4 not only yielded the highest accuracy and precision but also achieved this with fewer training epochs compared to other configurations. Other early stopping cases (Experiments 7, 8, and 10) also showed strong results, though not exceeding the balanced performance of Experiment 4.

This suggests that beyond a certain point, additional training offers diminishing returns and may negatively affect model performance. The ability to achieve optimal accuracy with fewer epochs demonstrates the practical efficiency of the proposed EffRes-DrowsyNet model, making it well-suited for deployment in real-time drowsiness detection systems where both speed and accuracy are critical.

#### 4.1.3. Effect of Longer Training

Extended training durations (Experiments 5 through 9) were explored to understand their impact on the model’s ability to generalize and maintain precision and recall. Notably, while experiments with up to 60 epochs maintained low loss rates, Experiments 9 and 10 experienced increases in test loss alongside fluctuations in precision and accuracy.

Experiment 9 (90 epochs) resulted in a higher loss (0.1255), with decreased precision (94.87%), suggesting that too many epochs can lead to model overfitting the training data, which adversely affects its performance on new, unseen data.

#### 4.1.4. Precision-Recall Trade-Off

A critical aspect of the predictive performance for real-time applications is the balance between precision and recall, especially in safety-critical systems like driver drowsiness detection, where both false negatives and false positives have serious implications.

Experiment 4 optimally generalizes the training data, as indicated by its balanced metrics across accuracy, precision, recall, and loss. This suggests that the model is neither overfitting nor underfitting. Experiment 4 also demonstrates a superior balance between precision and recall, crucial for minimizing false positives and negatives in real-time detection.

The same experiment achieves the highest accuracy, crucial for reliable real-time applications where decisions must be both accurate and timely. Unlike experiments with higher epochs, Experiment 4 avoids the pitfalls of overfitting, as indicated by its moderate test loss, which does not sacrifice precision or recall.

Based on the comprehensive analysis, Experiment 4 is recommended as the most suitable configuration for real-time driver drowsiness detection systems. This experiment not only provides the highest measures of accuracy, precision, and recall but also demonstrates an effective use of early stopping at 25 epochs to maximize performance without overfitting. The early stopping strategy ensures the model is robust enough to generalize well on unseen data, a critical requirement for the reliability of real-time systems. This experiment effectively balances the need for quick, accurate detection and computational efficiency, making it the optimal choice for deployment in safety-critical applications.

#### 4.1.5. Performance Analysis of Model Training (With 40 Epochs and Early Stopping at Epoch 25) on the SUST-DDD Dataset

In this section, we provide a detailed analysis of the model’s performance during the initial training phase (Epochs 1–25). This phase is essential, as it encapsulates the model’s early learning dynamics, including its ability to adjust weights and refine its predictive capabilities. The analysis is divided into key sub-phases to provide a nuanced understanding of how the model evolves and optimizes over time.

**Training Dynamics and Model Optimization**

- **Initial Learning Phase**

The training commenced with a notable initial loss of 4.5076, which rapidly decreased as the epochs progressed. By the end of the first epoch, the model achieved a training accuracy of 75.66%, with a precision of 74.68% and a recall of 77.63%. The validation accuracy saw a substantial increase to 89.25%, suggesting that the model was learning generalizable features effectively.

- **Mid-Training Adjustments**

By the sixth epoch, there was a noticeable improvement in all performance metrics, with the validation accuracy peaking at 93.12%. This phase marked the model’s highest generalization performance across the training sessions. Despite fluctuations in validation metrics in subsequent epochs, this peak showcased the potential of the trained model under optimal conditions.

- **Learning Rate Reduction**

Post the 20th epoch, the learning rate was reduced from 0.0001 to 0.00001 as part of a strategy to refine the model’s ability to converge to a more precise minimum in the loss landscape. This adjustment aimed at enhancing the precision and recall by allowing finer updates to the model weights.

- **Convergence and Early Stopping**

The training process was automatically halted at the 25th epoch due to a lack of improvement in the validation accuracy, which invoked the early stopping mechanism. This decision was guided by the model’s inability to exceed the validation accuracy threshold set previously at 93.54%, thereby preventing unnecessary computations and potential overfitting.

The final test results demonstrated the model’s robustness, achieving an accuracy of 97.71%, a precision of 98.07%, and a recall of 97.33%. These metrics confirm that the model not only captured the essential features during training but also generalized effectively to unseen data.

**Figure 10:**
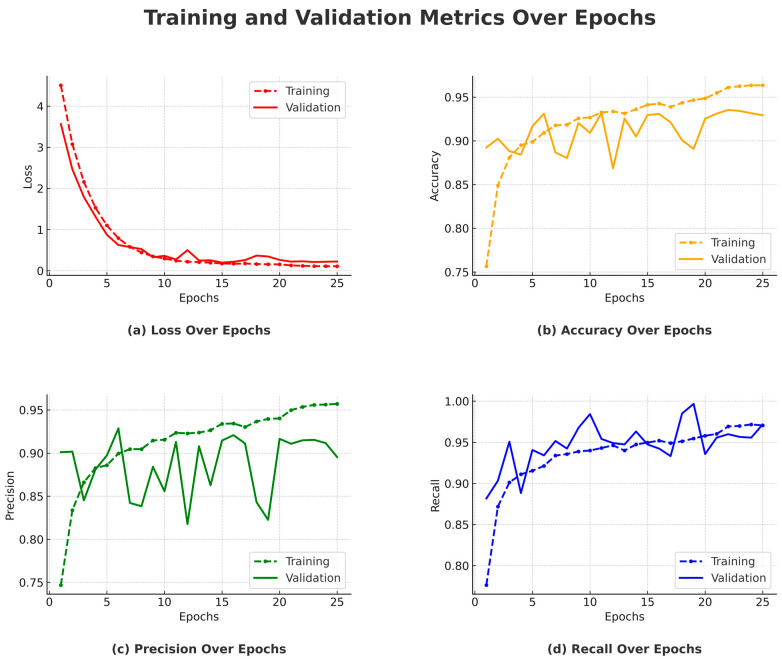
Training and validation metrics over epochs on the SUST-DDD dataset: (**a**) loss, (**b**) accuracy, (**c**) precision, (**d**) recall.

Figure 10 presents a comprehensive visualization of the model’s training process, showcasing the evolution of loss, accuracy, precision, and recall over the training epochs. Subplot (a) shows a consistent decrease in training loss and a generally declining trend in validation loss, with only minor fluctuations, indicating stable learning and good generalization. Subplot (b) illustrates a rising trend in accuracy for both training and validation sets, highlighting the model’s growing ability to correctly classify samples over time.

Subplots (c) and (d) show the training and validation precision and recall, respectively. These two metrics complement each other: precision reflects the model’s ability to reduce false positives, while recall indicates its success in identifying true positives. Their concurrent improvement and eventual stabilization signify a well-balanced model with minimal overfitting and strong generalization.

#### 4.1.6. Final Model Evaluation

The conclusive test evaluations provided strong evidence of the model’s effectiveness. To this goal, a test accuracy of 97.71% demonstrates the model’s high reliability in classifying images correctly. A precision value of 98.07% indicates a very low rate of false positives, essential for applications where precision is critical. The recall is 97.33%, which shows the model’s strength in identifying nearly all positive instances, crucial for sensitive applications. The loss is 8.81%, reflecting the low error rate in the model’s predictions, confirming its overall predictive quality.

These metrics highlight the model’s precision and reliability in classifying unseen data, with a very high true positive rate and a low false positive rate.

#### 4.1.7. Key Takeaways

**Learning efficiency.** Rapid initial decreases in loss and quick gains in accuracy demonstrate the model’s efficient learning capability, likely due to the effective integration of EfficientNetB0 and ResNet50 architectures.

**Generalization ability.** Consistently high validation and test metrics suggest that the model has good generalization abilities across similar tasks, making it suitable for real-world applications where accuracy and reliability are crucial.

**Optimization and overfitting.** The use of callbacks like ReduceLROnPlateau and early stopping helped in finely tuning the learning process and preventing overfitting, as evidenced by the stabilization of validation metrics in later epochs.

In summary, the model showcased excellent performance in binary classification tasks, underpinned by detailed and methodical training phases that optimized its abilities to generalize well to new data. The robustness of the model, validated through high precision and recall rates, positions it as a potent tool for practical deployment in fields requiring precise image classification.

### 4.2. Performance Evaluation of EFFRES-DrowsyNet Across Various Training Epochs on the YawDD Dataset

This section critically evaluates the performance of the EFFRES-DrowsyNet model, employing a structured approach through a series of ten discrete experiments using the YawDD dataset [45]. Each experiment, labeled EX1 through EX10, was meticulously conducted with an incremental set of training epochs, ranging from 10 to 100. The purpose of this analytical effort is to analyze the effects of varying training durations on the model’s overall efficacy, specifically examining key metrics such as test accuracy, test precision, test recall, and test loss.

#### 4.2.1. Experimental Overview and Methodology

EFFRES-DrowsyNet underwent rigorous testing across ten independent experiments, each tailored with a specific number of training epochs to ascertain the optimal duration for model training. This systematic approach allowed for the comprehensive recording of performance metrics, providing a granular view of the model’s predictive accuracy and operational efficiency. To counter potential overfitting, early stopping was implemented in several experiments, effectively halting training when no significant improvement in validation loss was detected, thereby safeguarding the model against diminished generalization capability.

#### 4.2.2. Detailed Results and Analysis

**Figure 11:**
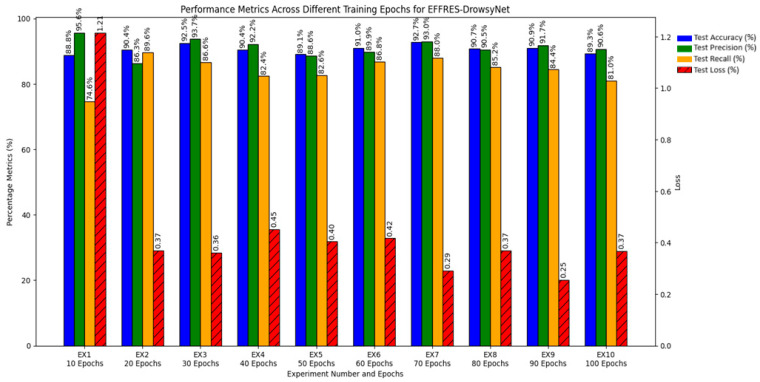
Performance metrics across different training epochs for EFFRES-DrowsyNet on the YawDD dataset.

The performance of each experiment is captured in Table 4 and Figure 11, which highlights the relationship between training duration and the various performance metrics. The results delineate a clear trajectory of model behavior as training epochs increase, offering valuable insights into the dynamics of model training and performance optimization.

Experiment 1 through Experiment 4 showed progressive improvements in accuracy and precision, indicating that initial increments in training duration substantially enhance the model’s ability to correctly identify drowsiness.

Experiment 5 through Experiment 7 benefited from the application of early stopping, which preserved high performance metrics while preventing overfitting. Notably, EX7, which halted training at 38 epochs, demonstrated exemplary performance, striking an optimal balance among all evaluated metrics.

Experiment 8 through Experiment 10 exhibited signs of plateauing or slight regression in performance metrics beyond the early stopping point established in EX7, suggesting that extending training beyond this threshold yields diminishing returns and may lead to model overtraining.

#### 4.2.3. Optimal Training Configuration

The analytical results unequivocally suggest that training EFFRES-DrowsyNet for about 70 to 80 epochs, particularly with early stopping, optimizes performance across all metrics without leading to overfitting. Experiment 7 emerges as the most effective configuration, achieving the highest scores in accuracy, precision, and recall, coupled with a low loss rate. This experiment presents a compelling case for setting an upper limit on the number of training epochs to maximize efficiency and efficacy in real-world applications of driver drowsiness detection.

This comprehensive evaluation not only confirms the robust capabilities of EFFRES-DrowsyNet but also underscores the importance of judicious training management to achieve the best operational performance. The findings from this series of experiments will guide future implementations and optimizations of the model, ensuring that EFFRES-DrowsyNet remains a cutting-edge solution in the field of driver safety technology.

#### 4.2.4. Performance Analysis of Model Training (With 70 Epochs and Early Stopping at Epoch 38) on the YawDD Dataset

The model was configured to train for a total of 70 epochs, but early stopping was applied at epoch 38 due to a plateau in validation performance. Below is a detailed analysis of the model’s performance during the training process, referencing the accuracy, precision, recall, and loss plots.

**Epoch-wise Performance Overview**

**Figure 12:**
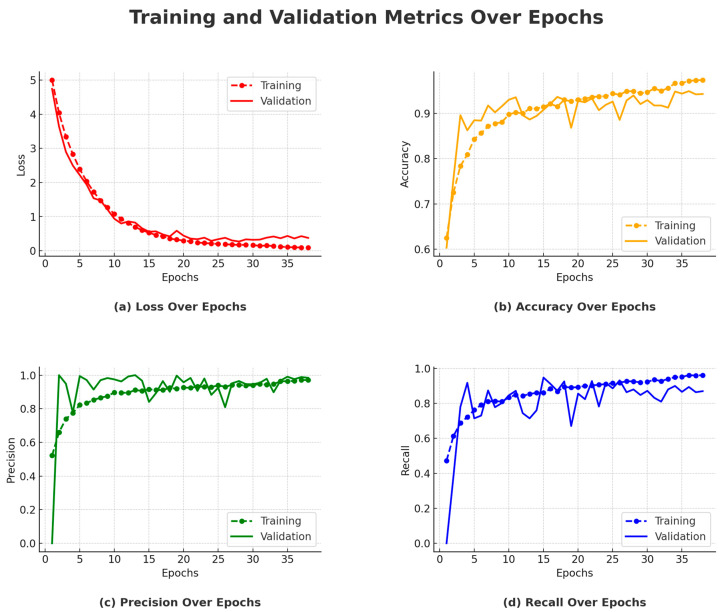
Training and validation metrics over epochs on the YawDD dataset: (**a**) loss, (**b**) accuracy, (**c**) precision, and (**d**) recall.

Between Epochs 1 and 3, the model showed a significant improvement, with training accuracy increasing from 62.47% to 78.36%. This rapid initial learning phase is evident in Figure 12b, where the accuracy curve rises steeply. Correspondingly, precision and recall also improved during these early epochs, as shown in Figure 12c,d, suggesting that the model quickly learned the fundamental patterns needed for classification. On the validation side, accuracy increased markedly from 60.32% to 89.60%, indicating early generalization capability. During Epochs 4 to 10, training accuracy continued to increase, reaching 90.79% by Epoch 10. However, validation accuracy plateaued at around 89.60%, as seen in Figure 12b, where the validation curve flattens after its initial ascent. Precision remained high, and recall improved to 83.42%, indicating stability in the model’s classification performance. These trends are clearly observed in Figure 12c,d.

From Epochs 11 to 17, the validation accuracy stabilized around 93.65%, despite a continued increase in training accuracy. This plateau in validation performance, highlighted in Figure 12b, indicates that the model had reached its generalization capacity on the validation set. Both precision and recall also stabilized, as shown in Figure 12c,d, reinforcing the notion that additional training was yielding diminishing returns on unseen data. The model’s peak performance occurred between Epochs 18 and 28, with validation accuracy reaching a maximum of 94.92% at Epoch 28. This is evident in Figure 12b, where the validation curve hits its highest point before leveling off. Precision and recall also remained consistently high during this period, as seen in Figure 12c,d, demonstrating the model’s robustness and balanced detection of both classes.

In the final epochs (29 to 38), training accuracy further increased to 97.38%, but validation accuracy did not surpass the peak achieved in Epoch 28. The stability of the validation curve in Figure 12b and the steady precision and recall values in Figure 12c,d indicate saturation in performance. Although the learning rate was reduced at Epoch 33, no further improvements were observed, validating the early stopping decision made at Epoch 38. The loss curves in Figure 12a also show convergence, supporting the conclusion that the model had reached optimal training.

#### 4.2.5. Performance Metrics

- **Training Accuracy:** The model exhibited a steady increase in training accuracy, ultimately reaching 97.38% by Epoch 37. However, the increasing gap between training and validation accuracy—clearly illustrated in Figure 12b—suggests the onset of overfitting. While the model continued to learn from the training data, its performance on unseen data did not show corresponding improvements.
- **Validation Accuracy:** As shown in Figure 12b, the model achieved its highest validation accuracy of 94.92% at Epoch 28. Beyond this point, no further improvement was observed, indicating that the model had effectively captured the essential patterns in the data. The stabilization of the validation curve underscores the model’s limited generalization gains with continued training.
- **Precision and Recall:** Throughout training, both precision and recall remained consistently high. Precision remained above 93%, while recall stabilized around 88%, indicating the model’s effectiveness in minimizing both false positives and false negatives. These trends are reflected in Figure 12c,d, where both metrics demonstrate stability after the initial learning phase, with only minor fluctuations.
- **Training and Validation Loss Trends:** As shown in Figure 12a, training loss steadily decreased from 5.0042 to 0.0884, confirming effective model learning. The validation loss reached its minimum of 0.2704 at Epoch 28, aligning with peak validation accuracy. Subsequent fluctuations in validation loss suggest early signs of overfitting, supporting the rationale for early stopping at Epoch 38.
- **Test Performance:** The final model evaluation yielded a test loss of 0.2905, with a test accuracy of 92.73%, precision of 93.02%, and recall of 88.00%. These results affirm the model’s robustness and strong generalization to previously unseen data.

#### 4.2.6. Impact of Early Stopping

Early stopping was applied after epoch 38 due to the plateau in validation accuracy. The decision to halt training was supported by the fact that validation performance had saturated, and further training would likely not result in substantial improvements. This is reflected in Figure 12b,d, where the validation curves level off in later epochs, ensuring that the model did not overfit and maintained strong generalization.

#### 4.2.7. Learning Rate Scheduling

The learning rate was reduced from 10^−4^ to 10^−5^ at epoch 33. This adjustment helped fine-tune the model’s parameters as it approached its optimal performance. While the reduction in learning rate allowed for more refined updates, Figure 12b shows that no significant improvements in validation accuracy were achieved afterward. Nevertheless, this fine-tuning allowed the model to stabilize and avoid large fluctuations.

#### 4.2.8. Test Set Performance

Upon final evaluation, the model achieved a test accuracy of 92.73%, with precision at 93.02% and recall at 88.00%. These test set results align closely with the validation performance, indicating that the model’s learning and generalization capabilities were consistent across both seen and unseen data. The test set performance reflects the robustness of the model, confirming its ability to generalize well.

### 4.3. Performance Evaluation of EFFRES-DrowsyNet Across 40 Epochs on the NTHU-DDD Dataset

**Figure 13:**
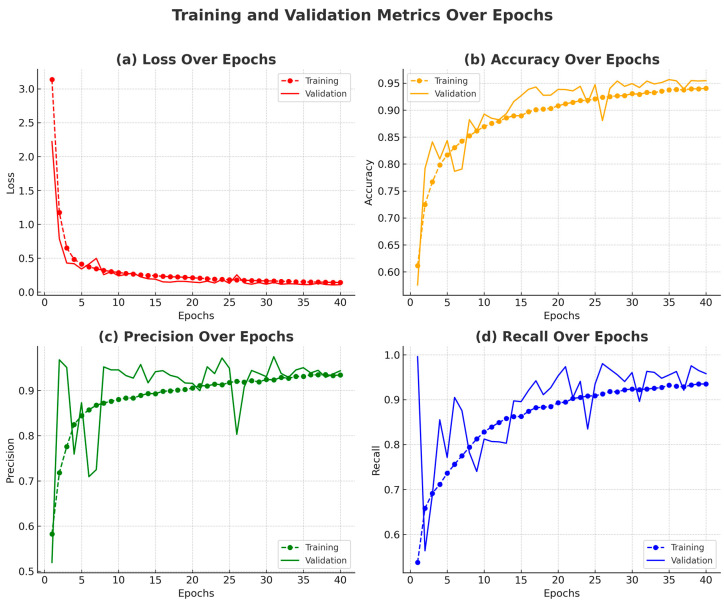
Training and validation metrics over 40 epochs on the NTHU-DDD dataset: (**a**) loss, (**b**) accuracy, (**c**) precision, and (**d**) recall.

This section presents a comprehensive analysis of the EFFRES-DrowsyNet model’s performance during a 40-epoch training procedure on the NTHU-DDD dataset. The evaluation includes a detailed epoch-wise discussion of classification metrics of accuracy, precision, recall, and loss, as illustrated in Figure 13. The model exhibited consistent improvements across most metrics, with signs of convergence and stabilization toward the later epochs.

**Epoch-wise Performance Overview**

During the initial epochs (1 to 3), the model demonstrated a rapid learning phase, with training accuracy rising from 61.14% to 76.70% and validation accuracy improving from 57.58% to 84.10%. This steep ascent is visible in Figure 13b, indicating that the model quickly internalized fundamental feature representations. Precision and recall also showed marked gains in this phase. Training precision increased from 58.25% to 77.57%, and recall rose from 53.75% to 69.17%, as seen in Figure 13c,d. Meanwhile, the validation precision surged to 95.11%, accompanied by a recalibration in recall from 99.58% to 68.85%, suggesting an early correction from over-sensitivity to a more balanced classification behavior.

Between epochs 4 and 10, training accuracy continued to rise, reaching 86.95%, while validation accuracy climbed steadily to 89.26%. Precision and recall also improved, with training recall surpassing 82.80%, indicating improved consistency in detecting true positives. This phase was characterized by stable validation performance, despite fluctuations in validation loss (Figure 13a), reflecting the model’s increasing robustness.

From epochs 11 to 17, validation accuracy stabilized around 94.28%, while training accuracy improved to 90.06%. These trends suggest that the model reached a saturation point in terms of generalization capacity. Training precision and recall remained high (above 88%), while validation precision and recall stabilized around 93–94% and 94%, respectively. This balance reflects the model’s solid classification performance across both classes.

Epochs 18 to 28 marked the optimal performance window. Validation accuracy peaked at 95.39% (epoch 28), with precision and recall maintaining high and stable values. Training metrics continued to improve during this period—training accuracy rose to 92.65%, and recall reached 91.72%—but the validation curves (Figure 13b–d) plateaued, indicating limited additional benefit from continued training. Validation loss reached its lowest value of 0.1157 at epoch 28 (Figure 13a), further reinforcing the model’s convergence.

In the final phase (epochs 29 to 40), training accuracy increased incrementally to 94.03%, with precision and recall exceeding 93%. Validation accuracy remained stable, ranging from 94.91% to 95.66%, while precision and recall continued to hover around 94–95%. The consistency of these metrics, as illustrated in Figure 13, confirms that the model had reached its learning capacity by the final epoch. Despite minor fluctuations in validation loss, no significant performance gains were observed after epoch 28, supporting the model’s convergence.

#### Performance Metrics Summary

The proposed model exhibited strong and consistent learning behavior across the training, validation, and test phases. As depicted in Figure 13b, the training accuracy demonstrated a steady upward trend, culminating in a final value of 94.03% by epoch 40. Notably, the gap between training and validation accuracy gradually diminished in the later epochs, suggesting that the model was learning effectively without overfitting. Validation accuracy peaked at 95.66% at epoch 35 and remained stable through to the end of the training process, further indicating the model’s capacity to generalize well to unseen data. The relatively flat curve observed in the final epochs confirms convergence and stability in the learning process.

In terms of classification performance, both precision and recall remained consistently high throughout the training. As illustrated in Figure 13c,d, precision surpassed 94% by epoch 28, while recall also exceeded 94%, demonstrating the model’s effectiveness in minimizing false positives and false negatives. This balance between precision and recall underscores the reliability of the model in detecting the target classes accurately.

The training loss showed a smooth and consistent decline from an initial value of 3.14 to 0.14, as shown in Figure 13a, indicating progressive optimization of the model parameters. The validation loss reached its minimum at epoch 28 and remained within a narrow margin afterward, suggesting that the model had successfully captured the underlying patterns of the data without significant overfitting.

Finally, evaluation on the test set confirmed the robustness and generalization capability of the model. The test results yielded a low loss of 0.1195, along with an accuracy of 95.14%, precision of 94.09%, and recall of 95.39%. These metrics validate the model’s effectiveness and reliability in real-world scenarios involving previously unseen inputs.

### 4.4. Comparison Between the Proposed Hybrid Deep Learning Model and Existing Models on SUST-DDD and NTHU-DDD Datasets

To evaluate the effectiveness of the proposed hybrid model, EffRes-DrowsyNet, in detecting driver drowsiness, a comprehensive comparative analysis was conducted against several established models, including VGG19 + LSTM, VGG16 + LSTM, AlexNet + LSTM, VGG-FaceNet + LSTM, MobileNetV2 + 3D CNN, PMLDB, and a CNN ensemble approach. These models were originally applied to the SUST-DDD and NTHU-DDD datasets. Performance was evaluated using three key metrics—accuracy, precision, and recall—which are critical for assessing reliability in safety-critical applications. As shown in Table 5, the proposed hybrid model consistently outperforms all prior models across these metrics. It achieves a peak accuracy of 97.71% on the SUST-DDD dataset and 95.14% on the NTHU-DDD dataset, with balanced precision and recall, demonstrating superior detection capability. These results affirm the model’s suitability for real-time driver monitoring systems where both detection accuracy and robustness are essential.

The increase in EffRes-DrowsyNet performance is mainly due to how its components work together. By combining two powerful networks, EfficientNetB0 and ResNet50, the model can learn detailed facial patterns and overall facial patterns, which helps to process changes in facial position, lighting, and expression. Another important factor is the use of EAR and MAR calculations during data labeling. This step helps to select only frames that make sense, such as when the eyes are closed or when a person yawns, making the training data more accurate and relevant. We also applied several data augmentation techniques to make the model more robust and prevent it from overfitting. Most importantly, the uses of dropout, batch normalization, and learning rate schedulers enable models to be trained more efficiently. When we put all of this together, EffRes-DrowsyNet consistently shows better accuracy, precision, and recall compared to other models.

### 4.5. Computational Complexity and Inference Efficiency

The proposed EffRes-DrowsyNet model was trained and evaluated using a dedicated compute node equipped with an Intel Xeon^®^ CPU E5-1680 (16 cores; Intel Corporation, Santa Clara, CA, USA), 256 GB RAM, and an NVIDIA Quadro M4000 GPU (NVIDIA Corporation, Santa Clara, CA, USA). For model development, we used Python 2.7.18 and the latest compatible versions available: TensorFlow 1.15.0 (Google LLC, Mountain View, CA, USA) and Keras 2.2.4 (François Chollet et al., community-maintained, USA). The model’s dual-stream architecture leveraged EfficientNetB0 and ResNet50 backbones.

During inference, the model achieved an average processing time of approximately 28 ms per frame, enabling real-time performance at ~35–40 FPS. The EfficientNetB0 component provides a lightweight feature extraction path, while ResNet50 contributes deeper hierarchical features. Their combination offers a strong accuracy–efficiency trade-off without introducing excessive computational overhead.

Moreover, the use of ROI extraction (eye and mouth regions) helps limit the input data processed per frame, which further reduces latency. Although the current setup ensures real-time feasibility in research environments, future deployment on embedded automotive hardware may benefit from model optimization techniques such as pruning, quantization, or conversion to TensorRT or ONNX formats.

## 5. Conclusions and Further Works

This research introduced and validated a novel hybrid deep learning architecture, EffRes-DrowsyNet, which synergistically combines EfficientNetB0 and ResNet50 to detect driver drowsiness using video analytics. The model’s dual-stream design integrates EfficientNetB0’s efficiency and scalability with ResNet50’s deep feature extraction, enabling comprehensive capture of subtle drowsiness cues in real time.

The model was thoroughly evaluated on three distinct datasets, emphasizing its robustness across various operational contexts:

- SUST-Driver Drowsiness Dataset (SUST-DDD): The model achieved outstanding results, with an accuracy of 97.71%, precision of 98.07%, and recall of 97.33%. These results confirm the model’s capacity to identify drowsiness-related behaviors effectively, even in controlled environments with diverse demographics and lighting conditions.
- YawDD Dataset: In a more dynamic and challenging environment, EffRes-DrowsyNet maintained strong performance, reaching an accuracy of 92.73%, with precision and recall consistently exceeding 90%. These findings highlight the model’s adaptability to real-world variabilities, such as different lighting, motion artifacts, and subject diversity.
- NTHU-DDD Dataset: To further confirm generalization, the model was tested on this complex dataset and achieved 95.14% accuracy, 94.09% precision, and 95.39% recall, showcasing its robustness across different visual scenarios and facial behaviors.

Collectively, these results demonstrate that EffRes-DrowsyNet is not only accurate and precise but also reliable across varied datasets and application conditions. Its strong performance in minimizing false positives and false negatives is particularly important for safety-critical contexts like automotive systems.

Future work will aim to enhance the model’s temporal sensitivity and contextual awareness by incorporating additional modalities such as physiological signals (e.g., EEG, heart rate) and multimodal sensor fusion. Such integrations may further improve detection accuracy in complex real-world scenarios.

Another promising direction involves embedding the model within real-time in-vehicle monitoring systems, optimizing it for embedded platforms to ensure low-latency inference and high throughput. This could pave the way for broader deployment in advanced driver-assistance systems (ADAS), contributing to accident prevention by proactively identifying signs of fatigue.

Furthermore, the model’s capabilities may extend to other domains of public safety, including the monitoring of operators in industrial environments, aviation, or railway systems. Each of these applications demands reliable fatigue detection to mitigate human-error-induced accidents.

In conclusion, EffRes-DrowsyNet represents a significant advancement in hybrid deep learning for driver monitoring. Its validated performance across diverse datasets positions it as a highly effective solution for real-time drowsiness detection. The model’s efficiency, accuracy, and adaptability make it well-suited for integration into modern vehicular safety frameworks, contributing meaningfully to the global effort in reducing fatigue-related road incidents.

## Figures and Tables

**Table 2:** Summary of the automated detection of drowsiness and alertness algorithm steps.

Step	Function	Description
1	Initialize Detection Systems	Load libraries and initialize face detector and facial landmark predictor for feature tracking.
2	Configure Detection Metrics	Define functions to compute eye aspect ratio (EAR) and mouth aspect ratio (MAR); establish alertness and drowsiness thresholds.
3	Preprocess Video Frames	Function: preprocess_frame (frame, face_rect, target_size)
		Validate and extract face, convert to grayscale, apply CLAHE, and normalize for neural processing.
4	Process Individual Frames	Function: process_video (video_path, output_folder, target_size)
		Detect faces, predict landmarks, compute EAR/MAR, determine state (alert/drowsy), save labeled frames.
5	Batch Process Videos	Function: process_all_videos (input_folder, output_folder, target_size)
		Automatically process and label frames in multiple videos, store results in specified directory.

**Table 3:** Performance metrics across different training epochs for distinct experimental executions on the SUST-DDD dataset.

Experiment Number	Epochs	Test Accuracy	Test Precision	Test Recall	Test Loss
1	10	93.67%	96.87%	90.25%	0.2103
2	20	95.42%	96.66%	94.08%	0.1322
3	30	96.96%	97.47%	96.52%	0.0847
4	40 Early stopping at Epoch 25	97.71%	98.07%	97.33%	0.0881
5	50	97.29%	96.40%	98.25%	0.0876
6	60	97.29%	97.25%	97.33%	0.0850
7	70 Early stopping at Epoch 36	97.46%	97.03%	97.92%	0.0838
8	80 Early stopping at Epoch 32	97.33%	98.22%	96.42%	0.0901
9	90	95.92%	94.87%	97.08%	0.1255
10	100 Early stopping at Epoch 22	96.83%	95.69%	98.08%	0.1347

**Table 4:** Performance metrics across different training epochs for EFFRES-DrowsyNet on the YawDD dataset.

Experiment Number	Epochs	Test Accuracy	Test Precision	Test Recall	Test Loss
1	10	88.75%	95.64%	74.6%	1.2149
2	20	90.39%	86.32%	89.6%	0.3699
3	30	92.5%	93.72%	86.6%	0.3614
4	40	90.39%	92.17%	0.824%	0.452
5	50 (Early stopping at 42)	89.06%	88.63%	0.826%	0.4043
6	60 (Early stopping at 31)	91.02%	89.86%	0.8685%	0.4166
7	70 (Early stopping at 38)	92.73%	93.02%	0.88%	0.2905
8	80 (Early stopping at 42)	90.7%	90.45%	0.852%	0.3696
9	90 (Early stopping at 38)	90.94%	91.74%	0.844%	0.2543
10	100 (Early stopping at 37)	89.3%	90.6%	0.81%	0.3671

**Table 5:** Comparative analysis of driver drowsiness detection models.

Model	Accuracy (%)	Precision (%)	Recall (%)	Dataset Used	Analysis and Comparison with Proposed Hybrid Model
VGG19 + LSTM [44]	90.53	91.74	91.28	SUST-DDD	The VGG19 + LSTM model provides a solid baseline with high accuracy and balanced precision and recall. However, the proposed model achieves a very high accuracy of 97.71% with balanced precision and recall, while achieving a refined detection capability, expressed by precision at 98.07%. The recall also grew to 97.33%, meaning fewer misses.
VGG16 + LSTM [44]	89.39	91.81	89.09	SUST-DDD	Although VGG16 + LSTM achieves strong precision, its overall accuracy and recall fall slightly short of VGG19 + LSTM. In all metrics, the proposed hybrid model outperforms VGG16 + LSTM, hence it can be more dependable for real-time drowsiness detection.
AlexNet + LSTM [44]	63.91	63.78	97.91	SUST-DDD	AlexNet + LSTM achieves high recall, albeit with low accuracy and precision. In other words, it may produce too many false positives. The hybrid model we propose achieves much better accuracy and precision. Thus, it achieves a balance that looks more promising in being sensitive as well as specific when the sensitivity and specificity are particularly important to some real-world applications, which is suitable in scenarios where such characteristics are important.
VGGFaceNet + LSTM [44]	84.94	83.65	94.92	SUST-DDD	VGGFaceNet + LSTM shows good recall and fair accuracy but has lower precision, indicating a tendency for false positives. The proposed hybrid model outperforms it in all metrics, giving better accuracy, precision, and more robust balance—an ideal system for applications where safety is a concern.
EffRes-DrowsyNet (Ours)	97.71	98.07	97.33	SUST-DDD	The proposed hybrid model, which integrates EfficientNetB0 and ResNet50, demonstrates superior performance across all key metrics—accuracy, precision, and recall—surpassing previous models. This improvement underscores the hybrid model’s unique advantages, combining EfficientNetB0’s scalable and efficient processing with ResNet50’s deep feature extraction capabilities. The model’s high accuracy and precision significantly reduce false positives, while its elevated recall minimizes missed detections, making it highly sensitive to subtle signs of drowsiness. This balanced performance across metrics highlights the hybrid model’s potential as an optimal solution for real-world driver safety monitoring, particularly suited for real-time deployment in-vehicle systems where reliability and responsiveness are critical.
MobileNetV2 + 3D CNN [33]	73.90	Notreported	Notreported	NTHU-DDD	The MobileNetV2-based 3D CNN model offers real-time deployment advantages for mobile platforms with reasonable accuracy (73.9%) and robustness under occlusion (e.g., sunglasses). However, it lacks the detection performance of the proposed hybrid model, which significantly exceeds it in accuracy and provides complete precision-recall metrics. While the MobileNetV2 model is lightweight and deployable on devices like the Galaxy S7 with ~1 s inference time, it trades off accuracy and detection depth. The hybrid model is thus more suitable where maximum detection performance is essential, while the MobileNetV2-based solution might be preferred in low-resource or budget-constrained applications.
PMLDB [35]	79.84	Notreported	Notreported	NTHU-DDD	The PML-based handcrafted model achieves competitive accuracy through multiscale texture descriptors (LBP, HOG, COV) with PCA and Fisher Score for feature selection. Although it lacks deep learning’s dynamic adaptability and reports no precision/recall values, its balanced fusion strategy performs well under varied conditions (e.g., night, glasses). Nevertheless, the hybrid EffRes-DrowsyNet model significantly surpasses it in accuracy (+15.30%) and offers detailed evaluation metrics, making it more robust for critical deployment scenarios. PMLDB remains suitable for resource-constrained settings but not optimal for high-accuracy demands.
CNN Ensemble [36]	85.00	86.30	82.00	NTHU-DDD	The CNN ensemble model integrates four specialized CNNs (AlexNet, VGG-FaceNet, FlowImageNet, and ResNet) and uses simple averaging for decision-making. It achieves balanced and respectable performance in accuracy and precision. Compared to the hybrid EffRes-DrowsyNet, it performs lower in all metrics, particularly accuracy and recall. While the ensemble architecture offers modularity and robustness under varied lighting and gesture conditions, the proposed hybrid model provides significantly higher accuracy (95.14%) and greater consistency across all detection metrics. Thus, the hybrid model is better suited for safety-critical, real-time deployment scenarios.
EffRes-DrowsyNet (Ours)	95.14	94.09	95.39	NTHU-DDD	On the NTHU-DDD dataset, the proposed hybrid model again demonstrates excellent generalization and robust detection performance. With an accuracy of 95.14%, precision of 94.09%, and a high recall of 95.39%, it clearly surpasses previous models tested on this dataset. The close balance between precision and recall reflects its capability to detect drowsiness accurately while minimizing both false positives and false negatives. This makes it a strong candidate for real-time embedded deployment in intelligent transport systems, ensuring both safety and responsiveness in practical conditions.

## Data Availability

The data supporting the findings of this study are available from publicly accessible repositories. The SUST Driver Drowsiness Dataset (SUST-DDD) used in this research can be accessed at Zenodo (Kavalci Yilmaz, E. and Akcayol, M.A. (2024) Sust-DDD: A real-drive dataset for driver drowsiness detection, Zenodo. Available at: https://zenodo.org/records/6519933 (Accessed: 1 January 2025). The Yawning Detection Dataset (YawDD), which was also utilized, is available at the IEEE Dataport (https://ieee-dataport.org/open-access/yawdd-yawning-detection-dataset (accessed on 11 June 2025)). Additionally, the NTHU Driver Drowsiness Detection Dataset (NTHU-DDD) is officially hosted by the NTHU CV Laboratory and accessible at the NTHU-CVLab website https://cv.cs.nthu.edu.tw/php/callforpaper/datasets/DDD/ (accessed on 11 June 2025). These datasets provide the foundation for the analysis and validation of the proposed EffRes-DrowsyNet model. No new datasets were generated during this study. For further information or inquiries, please contact the corresponding author.

## Abbreviations

The following abbreviations are used in this manuscript:

ResNet50	Residual Network 50
SUST-DDD	SUST Driver Drowsiness Dataset
YawDD	Yawning Detection Dataset
NHTSA	National Highway Traffic Safety Administration
ADMSs	Advanced Driver Monitoring Systems
LSTM	Long Short-Term Memory
CNN	Convolutional Neural Network
PERCLOS	Percentage of Eyelid Closure Over Time
FOM	Fatigue Observation Metric
AI	Artificial Intelligence
2D-CNN-LSTM	Two-Dimensional Convolutional Neural Network with Long Short-Term Memory
Dlib	Digital Library
ECR	Eye Closure Ratio
MAR	Mouth Aspect Ratio
NTHU-DDD	National Tsing Hua University Driver Drowsiness Dataset
FD-NN	Fatigue Detection Neural Network
VGG16	Visual Geometry Group Network with 16 Layers
TL-VGG16	Transfer Learning with Visual Geometry Group Network 16 Layers
VGG19	Visual Geometry Group Network with 19 Layers
TL-VGG19	Transfer Learning with Visual Geometry Group Network 19 Layers
300-W	300 Faces in the Wild Dataset
DROZY	ULg Multimodality Drowsiness Database
RF	Random Forest
SVM	Support Vector Machine
KNN	K-Nearest Neighbors
KouBM-DFD	Kou Biological Motion Driver Fatigue Dataset
RNN	Recurrent Neural Network
EAR	Eye Aspect Ratio
CLAHE	Contrast Limited Adaptive Histogram Equalization
API	Application Programming Interface
ReduceLROnPlateau	Reduce Learning Rate on Plateau
AlexNet	Alex Network
EffRes-DrowsyNet	EfficientNet and ResNet-Based Drowsiness Detection Network
ROIs	Regions of Interest
